# Machine learning redevelopment of GRACE, ACEF, and TIMI scores for 6-month mortality

**DOI:** 10.3389/frai.2026.1838324

**Published:** 2026-07-09

**Authors:** Bing Han, Zhaowei Zhu, Rui Guo, Jizhe Xu, Yixin Zhang, Zheng Zhang, Wenqiang Li

**Affiliations:** 1 Heart Center, The First Hospital of Lanzhou University, Lanzhou, China; 2 The First Clinical Medical School, Lanzhou University, Lanzhou, China; 3 Department of Cardiology, The Second Xiangya Hospital of Central South University, Changsha, China; 4 Department of Psychiatry, Qinghai Third People’s Hospital, Xining, China; 5 Department of Cardiology, Qinghai Cardio-Cerebrovascular Specialty Hospital, Xining, China

**Keywords:** acute myocardial infarction, machine learning, predictive model, risk score, six-month mortality

## Abstract

**Background:**

In recent years, advancements in our understanding of the pathophysiological mechanisms underlying coronary artery disease (CAD) have introduced new challenges regarding the clinical application of traditional risk scores. While studies suggest that machine learning (ML) algorithms surpass traditional statistical methods in risk prediction, their conclusions are often derived from heterogeneous datasets and varying model structures, which restrict their generalizability and persuasive power.

**Objective:**

This study aims to evaluate the clinical performance of the GRACE, ACEF, and TIMI risk scores, while also enhancing their predictive accuracy through redevelopment utilizing six ML algorithms.

**Methods:**

This retrospective study was undertaken at the First Hospital of Lanzhou University between January 1, 2019, and December 31, 2020. Six ML algorithms were employed to redevelop the original GRACE, TIMI, and ACEF risk scores. Model performance was evaluated using accuracy, sensitivity, precision, F1-score, the area under the receiver operating characteristic curve (AUROC), and the area under the precision-recall curve (AUPRC).

**Results:**

We retrospectively enrolled 1,682 patients diagnosed with acute myocardial infarction (AMI) who underwent emergency percutaneous coronary intervention (PCI). The derivation cohort from 2019 included 883 patients, among whom 48 (5.4%) experienced death during the follow-up period. The temporal validation cohort from 2020 consisted of 799 patients, with 26 (3.25%) reported deaths. The original GRACE, TIMI, and ACEF scores were validated using derivation data, resulting in AUROC values of 0.84, 0.68, and 0.69, and AUPRC values of 0.49, 0.19, and 0.34, respectively. The redevelopment of ML algorithms significantly enhanced predictive performance, with the ensemble algorithms yielding the most favorable results. The redeveloped GRACE, TIMI, and ACEF models achieved AUROC values of 0.89, 0.75, and 0.91, and AUPRC values of 0.55, 0.50, and 0.50, respectively. Demonstrating superior predictive performance for six-month mortality over the redeveloped ACEF and TIMI scores, the redeveloped GRACE score yielded an AUROC of 0.89 and an AUPRC of 0.32 in temporal validation.

**Conclusion:**

This study validated three established risk scores and found that the GRACE score exhibited superior predictive performance for six-month mortality compared to the ACEF and TIMI scores. The redevelopment of these scores using ML techniques significantly enhanced their predictive accuracy. This improvement reflects both the inherent strengths of ML algorithms and the recalibration of parameter weights to better align with contemporary clinical practice.

## Introduction

In recent years, advancements in understanding the pathophysiological mechanisms of coronary artery disease (CAD), along with the emergence of novel biomarkers, imaging technologies, and therapeutic strategies, have presented new challenges to the clinical application of traditional risk scores (Eagle et al., 2004; Ramsay et al., 2006; Tang et al., 2007). Moreover, in the context of unequal distribution of medical resources, a key issue remains how to implement simple and accurate risk scoring systems for the early identification and intervention of high-risk patients, particularly in under-resourced settings (Hung et al., 2021; Ösken et al., 2021). Although studies suggest that machine learning (ML) algorithms outperform traditional statistical methods in risk prediction (Mohd Faizal et al., 2021; Ravi et al., 2017), their conclusions are often based on heterogeneous datasets and model structures, which limit their generalizability and persuasiveness (Templin and Di Vece, 2021; Huang et al., 2016). Wenzl et al. (2022) reported that the GRACE 2.0 score demonstrated limited performance in predicting in-hospital mortality (IHM) among female patients with non-ST-elevation myocardial infarction (NSTEMI), often underestimating their risk. Utilizing the original eight parameters of the global registry of acute coronary events (GRACE) score, they applied the extreme gradient boosting (XGBoost) algorithm to develop GRACE 3.0, which improved performance across both sexes and reduced gender disparity in risk stratification. However, the model still fell short of achieving individualized risk prediction (Takahashi et al., 2020; Taniyama et al., 2020; Sabatine and Braunwald, 2021).

To systematically address these issues, this study first evaluated and compared the clinical performance of three established risk scores in patients with acute myocardial infarction (AMI): GRACE, age-creatinine-ejection fraction (ACEF), and thrombolysis in myocardial infarction (TIMI) (Chu et al., 2008; Huang et al., 2016; Gao et al., 2020; Meier et al., 2023). The GRACE score is currently recommended by guidelines for guiding intervention timing and predicting prognosis in AMI. In contrast, the TIMI score, which was developed earlier for risk stratification, emphasizes clinically accessible parameters, while the ACEF score is valued for its simplicity, ease of use, and robust predictive performance. Based on these comparative findings, we employed six ML algorithms to redevelop the original scores and identify the most effective modeling strategy. Finally, using the study dataset, we validated the three ML-redeveloped scores to quantify the impact of ML on model performance and compare their discriminative ability in predicting outcomes for AMI patients undergoing percutaneous coronary intervention (PCI). The main contributions of this study include: (1) validating the current clinical utility of the GRACE, ACEF, and TIMI scores; (2) comparing the performance of the three risk scores on a unified dataset; (3) exploring the enhancement of traditional scores through ML algorithms; and (4) confirming that model parameters and data quality are key determinants of predictive performance.

## Materials and methods

### Study population

**Figure 1:**
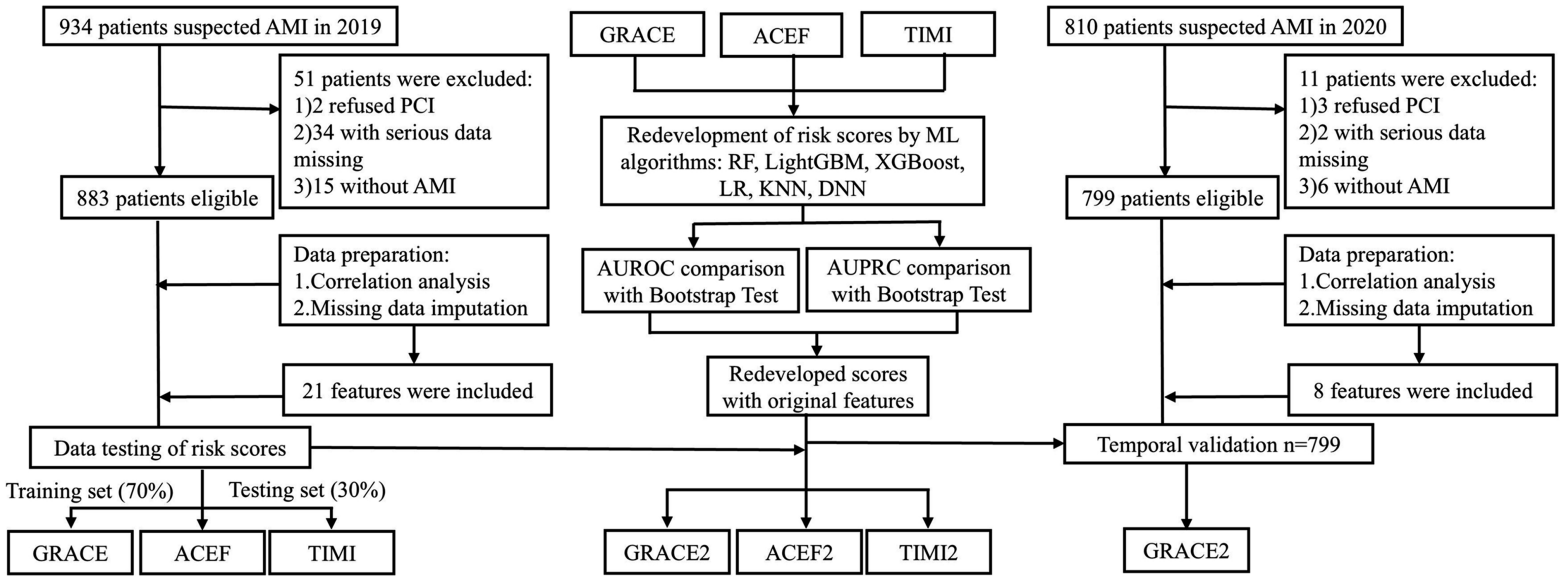
Flowchart of AMI patient selection and scores redevelopment process. GRACE, global registry of acute coronary events; TIMI, thrombolysis in myocardial infarction; ACEF, age-creatinine-ejection fraction; ML, machine learning; RF, random forest; LR, logistic regression; LightGBM, light gradient boosting machine; XGBoost, extreme gradient boosting; KNN, k-nearest neighbors; DNN, deep neural network; AUROC, area under the receiver operating characteristic curve; AUPRC, area under the precision-recall curve.

This single-center retrospective cohort study received ethical approval from the Ethics Committee of the First Hospital of Lanzhou University (Approval No. LDYYLL-2025-786). A total of 1,682 patients diagnosed with AMI who underwent emergency PCI at the Chest Pain Center of the First Hospital of Lanzhou University between January 1, 2019, and December 31, 2020, were retrospectively enrolled. Patients admitted in 2019 were designated as the derivation cohort, which was randomly divided into a training set (70%) and a testing set (30%). Meanwhile, patients admitted in 2020 were allocated to the temporal validation cohort (Figure 1). The diagnosis of AMI was based on criteria established by the European Society of Cardiology (Collet et al., 2021; Gaudino et al., 2023). The inclusion criteria were as follows: (1) confirmed AMI diagnosis, (2) age ≥18 years, (3) receipt of PCI, and (4) availability of complete clinical data. The inclusion and exclusion process were detailed in Figure 1. Follow-up data were obtained through structured telephone interviews conducted by a dedicated follow-up center, supplemented by outpatient clinical evaluations.

### Data collection

All patient data were collected through the electronic medical record system of the Cardiology Center at the First Hospital of Lanzhou University. Trained personnel were responsible for data entry, with dedicated staff overseeing data quality control and verification. Follow-up information was obtained through telephone interviews conducted by the PCI follow-up center and supplemented by outpatient visit records. The collected variables included age, mass, body mass index (BMI), type of acute myocardial infarction (AMI Type), anterior ST segment elevated myocardial infarction (Ant STEMI), ischemia time (IT), severe angina (SA), aspirin prior 7 days (ASA use), CAD, hypertension (HTN), type 2 diabetes mellitus (T2DM), hyperlipidemia (HLD), smoking history (SH), systolic blood pressure (SBP), heart rate (HR), creatinine (CREA), troponin I (TNI), left ventricular ejection fraction (LVEF), cardiac arrest (CA), ST segment deviation (ST Dev), Killip class (Killip), All laboratory data were derived from the first blood sample collected after admission, prior to emergency PCI. Echocardiographic parameters were based on the first ultrasound report generated within 24 h of admission. The primary endpoint of this study was all-cause mortality.

### Scores redevelopment and comparison

The GRACE, ACEF, and TIMI scores were evaluated using the study dataset to compare their predictive performance within the same population. Before redeveloping the three scores with ML, we performed a sample size estimation to enhance the reliability of the study results (see Supplementary material). Six ML algorithms: random forest (RF), LightGBM, XGBoost, logistic regression (LR), k-nearest neighbors (KNN), and deep neural networks (DNN) (Wang et al., 2021) were employed to optimize the original scores. To enhance model performance, Grid Search with Cross-Validation (GSCV) was utilized for hyperparameter tuning, systematically evaluating a variety of hyperparameter combinations to identify the optimal configuration. Model performance was assessed using bootstrap resampling to compare the AUROC and AUPRC values, thereby identifying the optimal algorithm for each score, which was subsequently reevaluated using the same derivation dataset (Zhou et al., 2021) (Figure 1).

### Statistical analysis

All statistical analyses and risk prediction models were developed utilizing Python version 3.11.9 software (Python Software Foundation, Wilmington, DE, USA). Continuous variables that followed a normal distribution were expressed as mean ± standard deviation and compared using the independent samples t-test. Non-normally distributed data were presented as median (Q1, Q3) and analyzed using the Mann–Whitney *U* test. The bootstrap hypothesis test was employed to evaluate the differences in the AUROC and the AUPRC between the models. We utilized a non-parametric bootstrap method with 1,000 replications to construct 95% bias-corrected confidence intervals (CI) for the indirect effect. Categorical variables were reported as counts (percentages) and compared using the chi-square test; if the expected frequency was less than 5, Fisher’s exact test was applied. A two-sided *p*-value of less than 0.05 was considered statistically significant.

## Results

### Population characteristics

This study included 883 AMI patients in 2019 who underwent PCI, of which 48 patients (5.4%) died during follow-up period. Regarding missing data, BMI was missing in 1 case (0.1%), SBP in 5 cases (0.6%), HR in 7 cases (0.8%), TNI in 6 cases (0.7%), and LVEF in 10 cases (1.1%). Since all missing rates were below 20%, missing values were imputed using multiple imputation by chained equations (MICE). Significant baseline differences were observed between the survived and deceased cohorts, including age, T2DM, SBP, HR, severe angina, CREA, TNI, LVEF, CA, Killip class I, and Killip class IV (*p* < 0.05). Of the total 883 cases, 618 (70%) were randomly assigned to the training cohort and 265 (30%) to the testing cohort, with no significant baseline differences between the two groups (Table 1). However, comparison between the derivation cohort and the temporal validation cohort revealed significant differences in HR, ST deviation, and mortality (*p* < 0.05) (Table 2).

**Table 1:** Baseline characteristics of patients in the survived, deceased, training and testing sets.

Variables	Survived (*n* = 835)	Deceased (*n* = 48)	Training (*n* = 618)	Testing (*n* = 265)
Age (y)	61.05 ± 11.08	65.83 ± 12.22^**^	61.30 ± 11.43	61.32 ± 10.64
T2DM (%)	148 (17.72)	16 (33.33)^**^	109 (17.63)	55 (20.75)
HTN (%)	406 (48.62)	24 (50)	306 (49.51)	124 (46.79)
HLD (%)	48 (5.75)	1 (2.08)	31 (5.02)	18 (6.79)
CAD (%)	83 (9.94)	6 (12.5)	64 (10.36)	25 (9.43)
SH (%)	500 (59.88)	25 (52.08)	370 (59.87)	155 (58.49)
Ant STEMI (%)	433 (51.86)	25 (52.08)	321 (59.94)	137 (51.70)
AMI Type (%)	636 (76.17)	36 (75.00)	471 (76.21)	201 (75.85)
SA (%)	208 (24.91)	19 (39.58)^*^	153 (24.76)	74 (27.94)
ASA use (%)	75 (8.98)	4 (8.33)	56 (9.01)	23 (8.68)
Mass (Kg)	69.18 ± 11.04	66.87 ± 11.86	69.09 ± 11.48	68.96 ± 10.15
BMI (Kg/m^2^)	24.30 ± 3.42	23.57 ± 3.68	24.24 ± 3.52	24.29 ± 3.26
IT (h)	13.81 ± 40.53	18.92 ± 37.33	13.58 ± 42.08	15.28 ± 36.05
SBP (mmHg)	117.28 ± 24.56	105.90 ± 29.80^**^	116.39 ± 24.81	117.30 ± 25.44
HR (beats/min)	81.40 ± 17.82	89.96 ± 22.54^**^	81.48 ± 18.86	82.78 ± 16.56
CREA (μmol/L)	72 (64, 84)	111 (85.5, 146)^***^	83.23 ± 70.96	85.14 ± 66.55
TNI (ng/mL)	1.8 (0.32, 7.3)	2.85 (0.95, 14.44)^*^	6.00 ± 8.34	5.60 ± 8.12
LVEF (%)	52.08 ± 6.86	43.87 ± 8.85^***^	51.83 ± 7.24	51.71 ± 7.18
CA	17 (2.04)	13 (27.08)^***^	22 (3.56)	8 (3.02)
ST Dev (%)	636 (76.17)	36 (75)	471 (76.21)	201 (75.85)
Killip1 (%)	760 (91.02)	24 (50)^***^	551 (89.16)	233 (87.92)
Killip2 (%)	51 (6.11)	3 (6.25)^***^	37 (5.99)	17 (6.41)
Killip3 (%)	14 (1.68)	1 (2.08)^***^	10 (1.62)	5 (1.89)
Killip4 (%)	10 (1.20)	20 (41.67)***	20 (3.24)	10(3.77)
Deceased (%)	0 (0)	48 (100)	33 (5.33)	15 (5.66)

Compared to survived, ^*^p < 0.05; ^**^p < 0.01; ^***^p < 0.001.

Continuous values were presented as median ± standard deviation. Categorical values were presented as number (percentage).

CAD, coronary artery disease; HTN, hypertension; T2DM, type 2 diabetes mellitus; HLD, hyperlipidemia; SH, smoking history; BMI, body mass index; SBP, systolic blood pressure; HR, heart rate; CREA, serum creatinine; TNI, troponin I; LVEF, left ventricular ejection fraction; CA, cardiac arrest; ST Dev, ST segment deviation; AMI Type, type of acute myocardial infarction; Ant STEMI, anterior ST segment elevated myocardial infarction; IT, ischemia time; SA, severe angina; ASA use, aspirin prior 7 days.

**Table 2:** Baseline characteristics of the derivation and temporal validation cohorts.

Variables	Derivation cohort AMI2019 (*n* = 883)	Temporal validation cohort AMI2020 (*n* = 799)	*p* value
Age (y)	61.31 ± 11.19	60.54 ± 11.06	0.157
SBP (mmHg)	116.66 ± 24.99	115.34 ± 26.63	0.293
HR (beats/min)	81.87 ± 18.20	79.94 ± 17.10^*^	0.025
CREA (μmol/L)	83.80 ± 69.63	78.41 ± 46.55	0.065
TNI (ng/mL)	5.88 ± 8.27	7.45 ± 26.20	0.092
CA	30 (3.40)	19 (2.38)	0.275
ST Dev (%)	672 (76.10)	654 (81.85)^**^	0.005
Killip1 (%)	784 (88.79)	684 (85.71)	0.159
Killip2 (%)	54 (6.12)	55 (6.89)	0.159
Killip3 (%)	15 (1.70)	15 (1.88)	0.159
Killip4 (%)	30 (3.40)	44 (5.51)	0.159
Deceased (%)	48 (5.44)	26 (3.25)^*^	0.039

Compared to AMI2019, ^*^p < 0.05; ^**^p < 0.01; ^***^p < 0.001.

Continuous values were presented as median ± standard deviation. Categorical values were presented as number (percentage).

SBP, systolic blood pressure; HR, heart rate; CREA, serum creatinine; TNI, troponin I; LVEF, left ventricular ejection fraction; CA, cardiac arrest; ST Dev, ST segment deviation.

### Comparison of GRACE, ACEF, and TIMI scores

**Figure 2:**
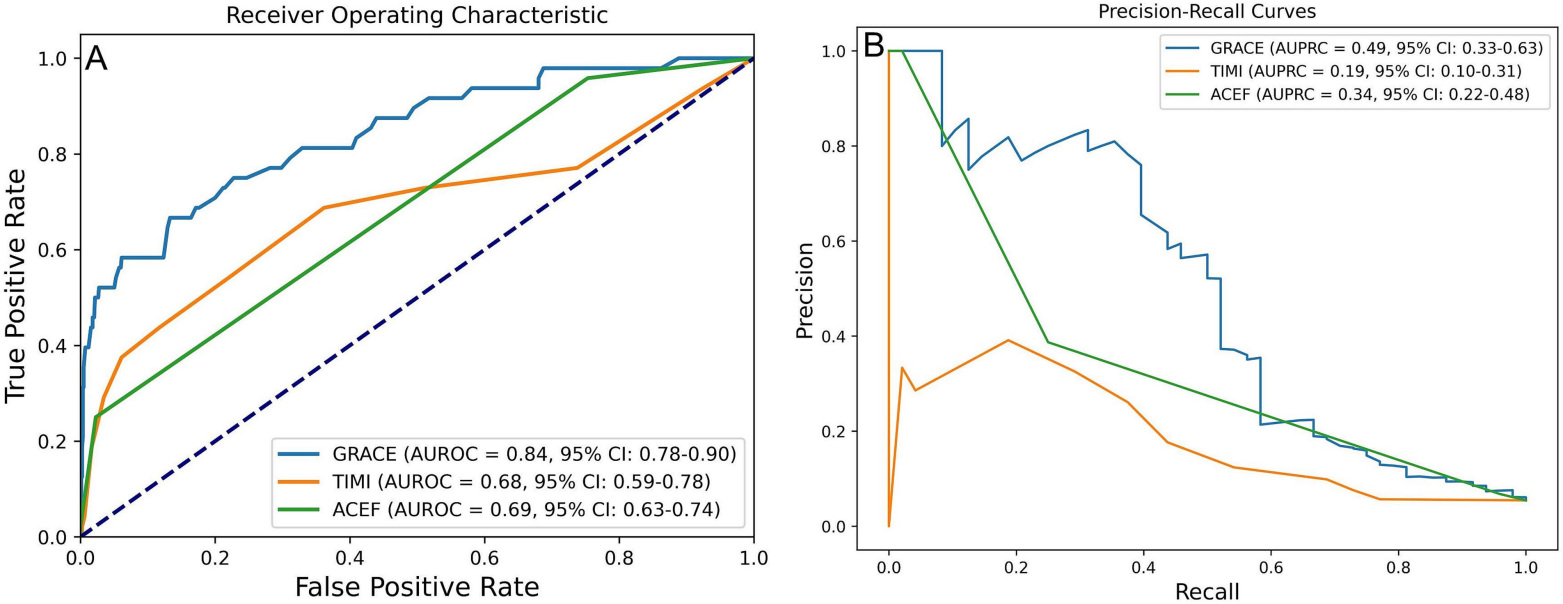
Predictive performance comparison of GRACE, ACEF, and TIMI scores for 6-month mortality in AMI patients post-PCI. GRACE, global registry of acute coronary events; TIMI, thrombolysis in myocardial infarction; ACEF, age, creatinine, ejection fraction; AMI, acute myocardial infarction; PCI, percutaneous coronary intervention; AUROC, area under the receiver operating characteristic curve; AUPRC, area under the precision-recall. **(A)** AUROC values of the GRACE, TIMI, ACEF scores. **(B)** AUPRC values of the GRACE,TIMI, ACEF scores.

The predictive performance of the GRACE, ACEF, and TIMI scores was evaluated, as illustrated in Figure 2. Detailed scoring criteria are presented in Supplementary Table S1. The GRACE score exhibited the highest performance, with an AUROC of 0.84 (95% CI: 0.78–0.90) and an AUPRC of 0.49 (95% CI: 0.33–0.63). In contrast, the ACEF score demonstrated a lower performance, with an AUROC of 0.69 (95% CI: 0.63–0.74) and an AUPRC of 0.34 (95% CI: 0.22–0.48). The TIMI score displayed the lowest performance among the three, with an AUROC of 0.68 (95% CI: 0.59–0.78) and an AUPRC of 0.19 (95% CI: 0.10–0.31).

The performance of the GRACE, ACEF, and TIMI scores was evaluated through various metrics, including accuracy, precision, sensitivity, specificity, F1 score, AUROC, and AUPRC, as presented in Table 3.

**Table 3:** Performance of GRACE, ACEF, TIMI score.

Score	Accuracy	Precision	Sensitivity	Specificity	F1-score	AUROC	AUPRC
GRACE	0.949	0.533	0.500	0.975	0.516	0.842	0.487
ACEF	0.938	0.387	0.250	0.977	0.304	0.686	0.342
TIMI	0.502	0.076	0.729	0.489	0.137	0.683	0.186

GRACE, global registry of acute coronary events; TIMI, thrombolysis in myocardial infarction; ACE, age-creatinine-ejection fraction; AUROC, area under the receiver operating characteristic curve; AUPRC, area under the precision-recall.

### Redevelopment of the GRACE score

**Figure 3:**
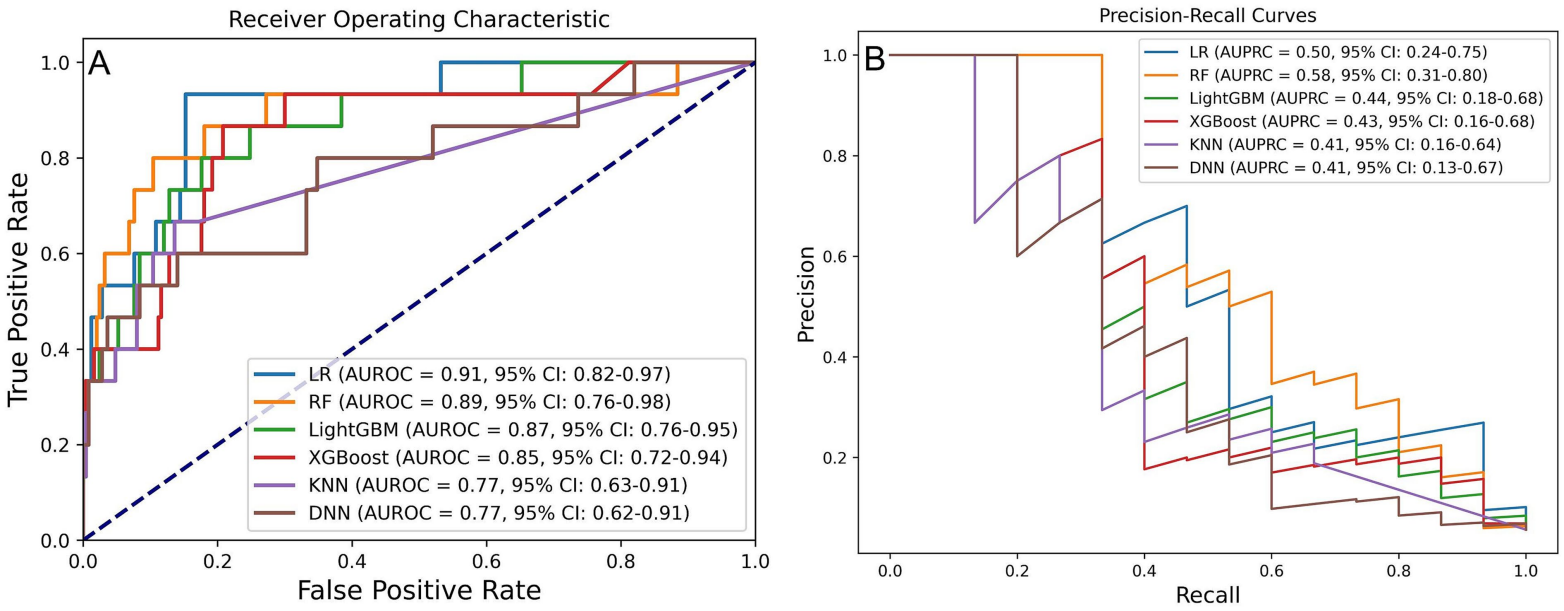
The GRACE score redevelopment for six-month mortality in AMI patients post-PCI. **(A)** AUROC values of redeveloped GRACE scores. **(B)** AUPRC values of redeveloped GRACE scores. GRACE, global registry of acute coronary events; AMI, acute myocardial infarction; PCI, percutaneous coronary intervention; RF, random forest; LR, logistic regression; LightGBM, light gradient boosting machine; XGBoost, extreme gradient boosting; KNN, k-nearest neighbors; DNN, deep neural network; AUROC, area under the receiver operating characteristic curve; AUPRC, area under the precision-recall curve.

Six ML algorithms were employed to redevelop the GRACE score, with model performance assessed using the AUROC and the AUPRC (Figure 3). In the validation set, LR achieved an AUROC of 0.91 (95% CI, 0.82–0.97) and an AUPRC of 0.50 (95% CI, 0.24–0.75). In contrast, the RF algorithm yielded an AUROC of 0.89 (95% CI, 0.76–0.98) and an AUPRC of 0.58 (95% CI, 0.31–0.80).

Bootstrap test was utilized to evaluate the differences in AUROC and AUPRC among the redeveloped GRACE scores (Supplementary Figure S1). The findings indicated the LR model demonstrated superior performance compared to XGBoost, KNN models, and LightGBM model demonstrated superior performance compared to KNN model, with statistically significant differences in AUROC (*p* < 0.05). In the AUPRC comparison, the RF model demonstrated superior performance compared to XGBoost, KNN and DNN models, the LR model demonstrated superior performance compared to DNN model, with statistically significant differences observed (*p* < 0.05).

To assess the contribution of the eight features included in the redeveloped GRACE model for predicting six-month mortality risk in AMI patients post-PCI, this study employed SHAP analysis to generate a feature importance scatter plot (Supplementary Figure S2). The features were ranked by their contribution to the model in the following order: Killip, CREA, Age, SBP, HR, CA, TNI, and ST Dev.

The feature radar chart of the redeveloped GRACE model, which predicts six-month mortality risk in AMI patients post-PCI, illustrates the feature names and their weight rankings (Supplementary Figure S3A). In addition, the feature bar chart presents the rankings and relative weight values of these features (Supplementary Figure S3B). Seven features with weights exceeding 0.02 were ranked as follows: Killip (0.274), CREA (0.181), CA (0.125), SBP (0.118), HR (0.099), Age (0.094), and TNI (0.094).

### Redevelopment of the ACEF score

**Figure 4:**
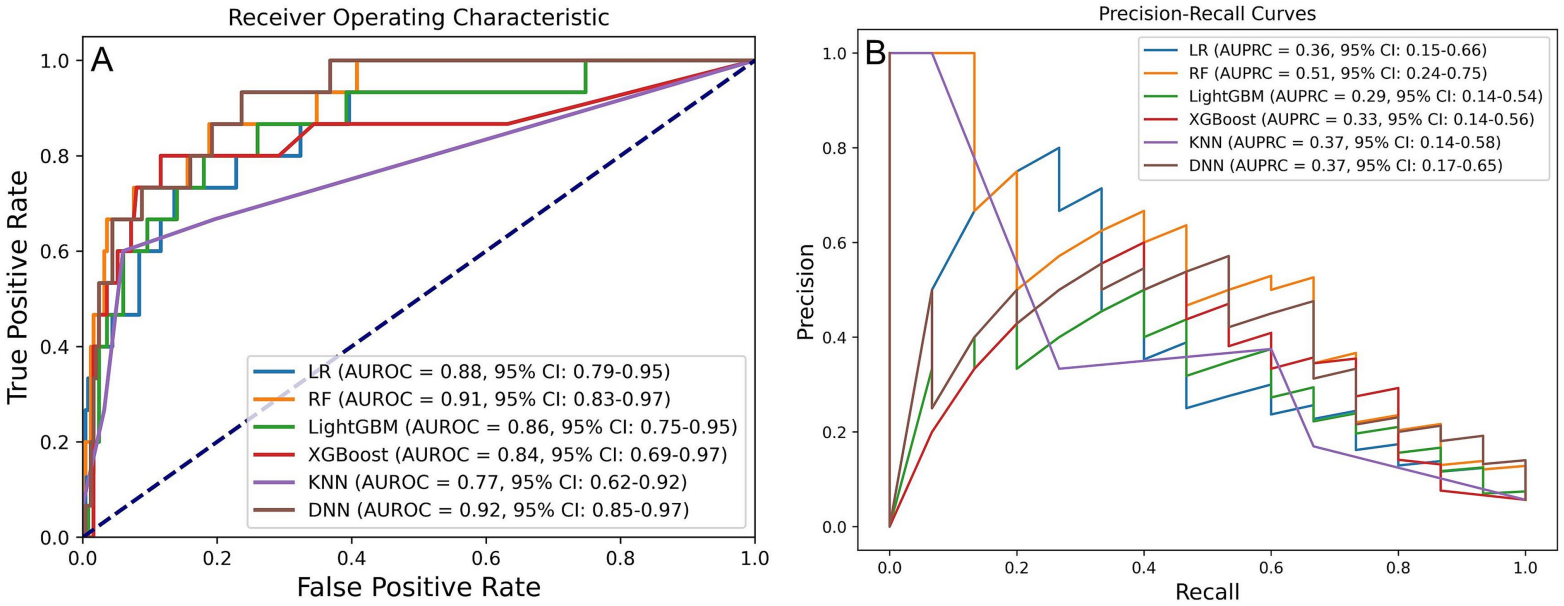
The ACEF score redevelopment for six-month mortality in AMI patients post-PCI. **(A)** AUROC values of redeveloped ACEF scores. **(B)** AUPRC values of redeveloped ACEF scores. ACEF, age-creatinine-ejection fraction; ML, machine learning; AMI, acute myocardial infarction; PCI, percutaneous coronary intervention; RF, random forest; LR, logistic regression; LightGBM, light gradient boosting machine; XGBoost, extreme gradient boosting; KNN, k-nearest neighbors; DNN, deep neural network; AUROC, area under the receiver operating characteristic curve; AUPRC, area under the precision-recall curve.

Six ML algorithms were employed to redevelop the ACEF score, with the AUROC and AUPRC metrics utilized to assess the predictive performance of the redeveloped ACEF scores (Figure 4). In the validation set, the RF algorithm demonstrated an AUROC of 0.91 (95% CI, 0.83–0.97) and an AUPRC of 0.51 (95% CI, 0.24–0.75).

A bootstrap test was conducted to compare the differences in AUROC and AUPRC among the redeveloped ACEF scores (Supplementary Figure S4). The findings indicated that the RF model exhibited superior performance compared to the XGBoost, KNN, and DNN models. Additionally, the LR model outperformed the KNN model, with statistically significant differences in AUROC (*p* < 0.05). In the AUPRC comparison, the RF model also demonstrated superior performance relative to the LightGBM model, with statistically significant differences observed (*p* < 0.05).

To assess the contribution of the three features included in the redeveloped ACEF for predicting six-month mortality risk in AMI patients post-PCI, this study employed SHAP analysis to generate a feature importance scatter plot (Supplementary Figure S5). The features were ranked by their contribution to the model in the following order: CREA, LVEF, and age.

The feature radar chart of the redeveloped ACEF model, which predicts six-month mortality risk in AMI patients post-PCI, illustrates the feature names and their weight rankings (Supplementary Figure S6A). In addition, the feature bar chart presents the rankings and relative weight values of these features (Supplementary Figure S6B). The three features included in the model are ranked as follows: CREA (0.412), LVEF (0.386), and Age (0.202).

### Redevelopment of the TIMI score

**Figure 5:**
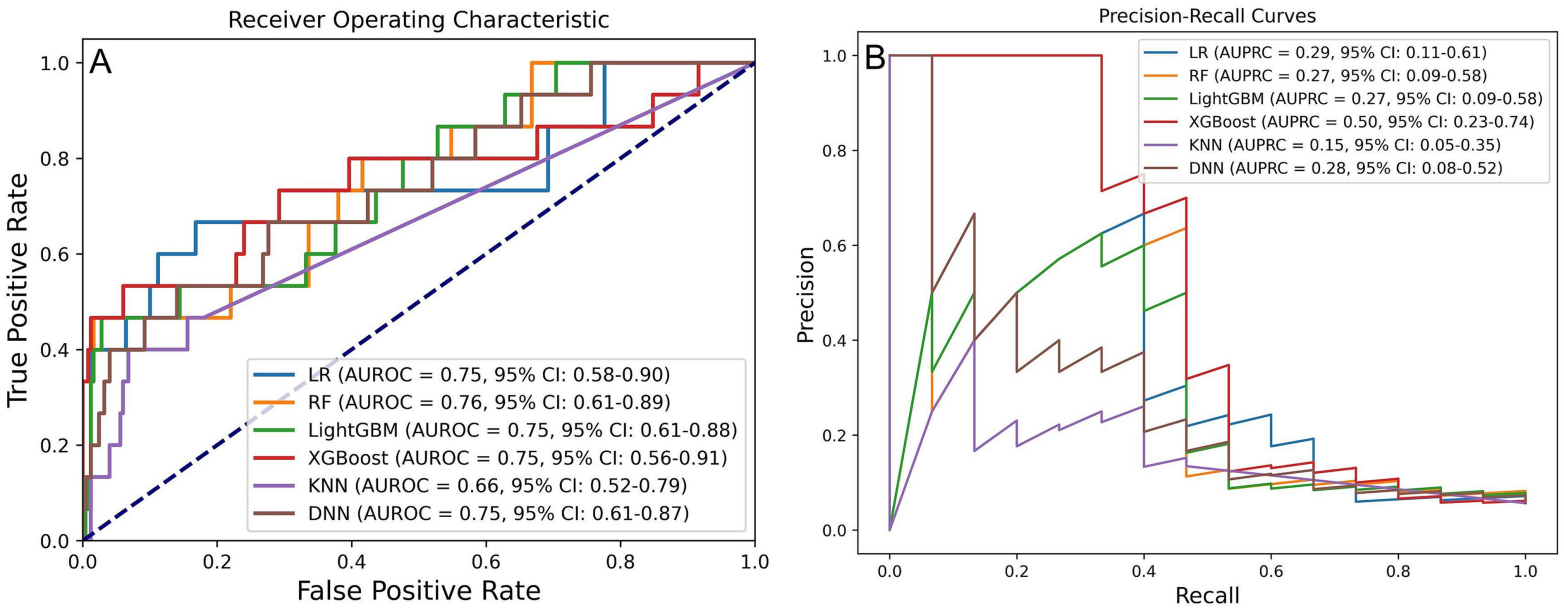
The TIMI score redevelopment for 6-month mortality in AMI patients post-PCI. **(A)** AUROC values of redeveloped TIMI scores. **(B)** AUPRC values of redeveloped TIMI scores. TIMI, thrombolysis in myocardial infarction; AMI: acute myocardial infarction; PCI: percutaneous coronary intervention; RF, random forest; LR, logistic regression; LightGBM, light gradient boosting machine; XGBoost, extreme gradient boosting; KNN, k-nearest neighbors; DNN, deep neural network; AUROC, area under the receiver operating characteristic curve; AUPRC, area under the precision-recall curve.

The predictive performance of the redeveloped TIMI score was assessed using AUROC and AUPRC across six ML algorithms (Figure 5). In the validation set, the XGBoost algorithm yielded an AUROC of 0.75 (95% CI: 0.56–0.91) and an AUPRC of 0.50 (95% CI: 0.23–0.74).

A bootstrap test was conducted to compare the differences in AUROC and AUPRC among the redeveloped TIMI scores (Supplementary Figure S7). The results indicated that the XGBoost model demonstrated superior performance compared to the RF, LightGBM and KNN models, the LR, RF models demonstrated superior performance compared to the KNN model, with statistically significant differences observed in AUPRC (*p* < 0.05).

To evaluate the contribution of all the features incorporated in the redeveloped TIMI for predicting six-month mortality risk in AMI patients post-PCI, this study utilized SHAP analysis to produce a feature importance scatter plot (Supplementary Figure S8). The features were ranked by their contribution to the model in the following order: Killip, IT, Mass, Age, TNI, BMI, Ant STEMI, SH, AMI Type, T2DM, HTN, SA, CAD, ASA use, HLD.

The feature radar chart of the redeveloped TIMI model, which predicts six-month mortality risk in AMI patients post-PCI, illustrates the feature names and their weight rankings (Supplementary Figure S9A). In addition, the feature bar chart presents the rankings and relative weight values of these features (Supplementary Figure S9B). The nine features with weights exceeding 0.05 included in the model are ranked as follows: Killip (0.354), Mass (0.076), T2DM (0.075), IT (0.061), HTN (0.059), Age (0.057), AMI Type (0.052), BMI (0.052), TNI (0.052).

### Comparison of the GRACE2, ACEF2, and TIMI2 scores

**Figure 6:**
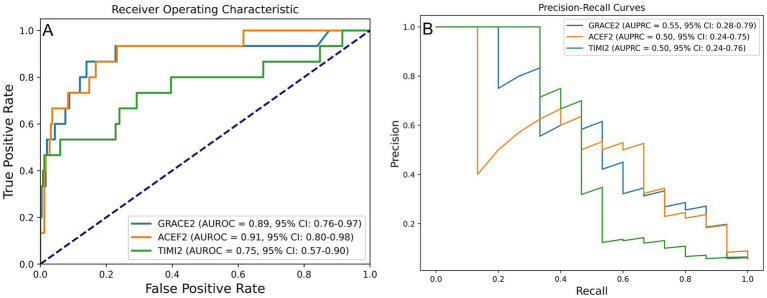
Predictive performance comparison of the GRACE2, ACEF2, and TIMI2 scores for 6-month mortality in post-PCI AMI patients. AMI, acute myocardial infarction; PCI, percutaneous coronary intervention; AUROC, area under the receiver operating characteristic curve; AUPRC, area under the precision-recall. **(A)** AUROC values of the GRACE2, TIMI2, ACEF2 scores. **(B)** AUPRC values of the GRACE2, TIMI2, ACEF2 scores.

The predictive performance of the redeveloped GRACE, ACEF, and TIMI scores was evaluated, as illustrated in Figure 6. The ACEF2 score demonstrated the highest performance, achieving an AUROC of 0.91 (95% CI: 0.80–0.98) and an AUPRC of 0.50 (95% CI: 0.24–0.75). The GRACE2 score exhibited comparable performance, with an AUROC of 0.89 (95% CI: 0.76–0.97) and an AUPRC of 0.55 (95% CI: 0.28–0.79). In contrast, the TIMI2 score showed the lowest performance among the three, with an AUROC of 0.75 (95% CI: 0.57–0.90) and an AUPRC of 0.50 (95% CI: 0.24–0.76).

**Figure 7:**
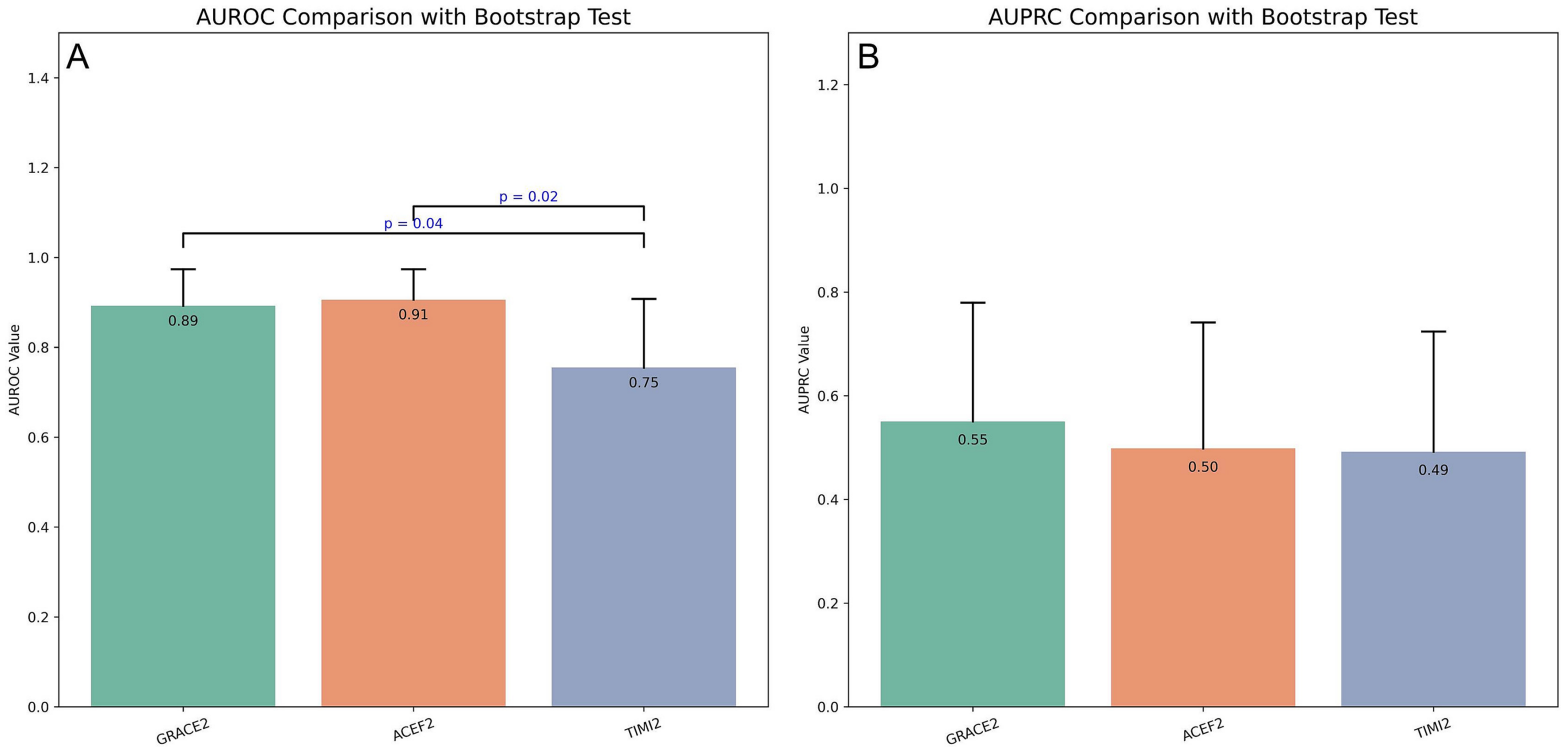
Comparison of GRACE2, ACEF2, TIMI2 scores using bootstrap test. **(A)** Bootstrap test for AUROC comparison. **(B)** Bootstrap test for AUPRC comparison. AUROC, area under the receiver operating characteristic curve; AUPRC, area under the precision-recall.

A bootstrap test was employed to assess the differences in the AUROC and the AUPRC among the GRACE2, ACEF2, and TIMI2 scores (Figure 7). The results indicated that the GRACE2 and ACEF2 exhibited superior performance relative to the TIMI2, with statistically significant differences in AUROC (*p* < 0.05). In the AUPRC comparison, the GRACE2 showed superior performance compared to the others, without statistically significant differences (*p* > 0.05).

The GRACE2 and ACEF2 scores demonstrated superior predictive performance compared to the redeveloped TIMI score; however, no statistically significant difference was found between the GRACE2 and ACEF2 scores. In accordance with the principle of minimal optimality, the ACEF2 score is deemed the most suitable option.

**Figure 8:**
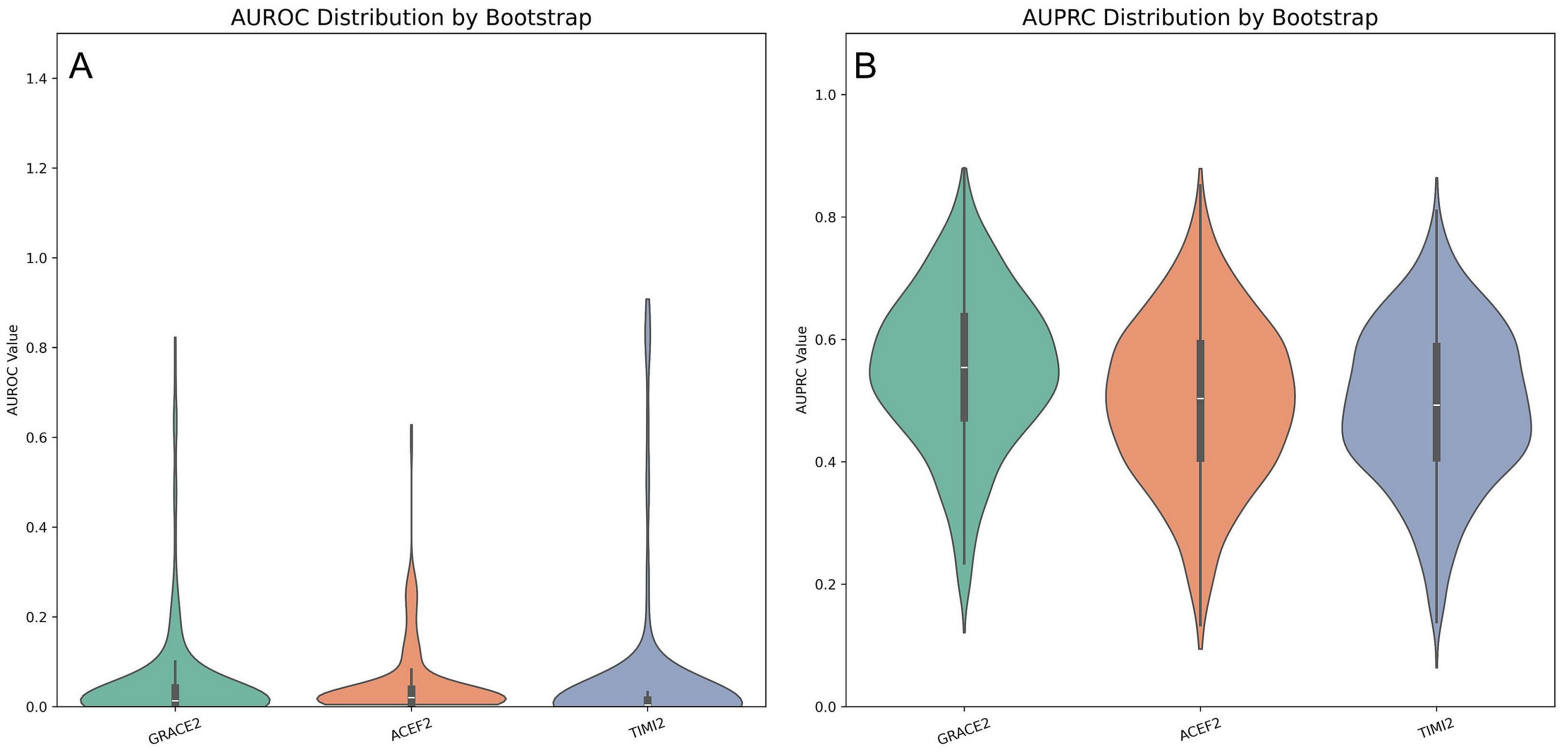
Violin plots comparing performance metrics across the GRACE2, ACEF2, TIMI2 scores. **(A)** AUROC distribution of the three scores. **(B)** AUPRC distribution of the three scores.

Violin plots were employed to visualize the probability density distributions of AUROC and AUPRC (Figure 8). The GRACE2 exhibited a more concentrated AUROC distribution compared to ACEF2 (Figure 8A). Its AUPRC distribution was right-skewed, with a median around 0.6, indicating superior discriminative ability.

**Figure 9:**
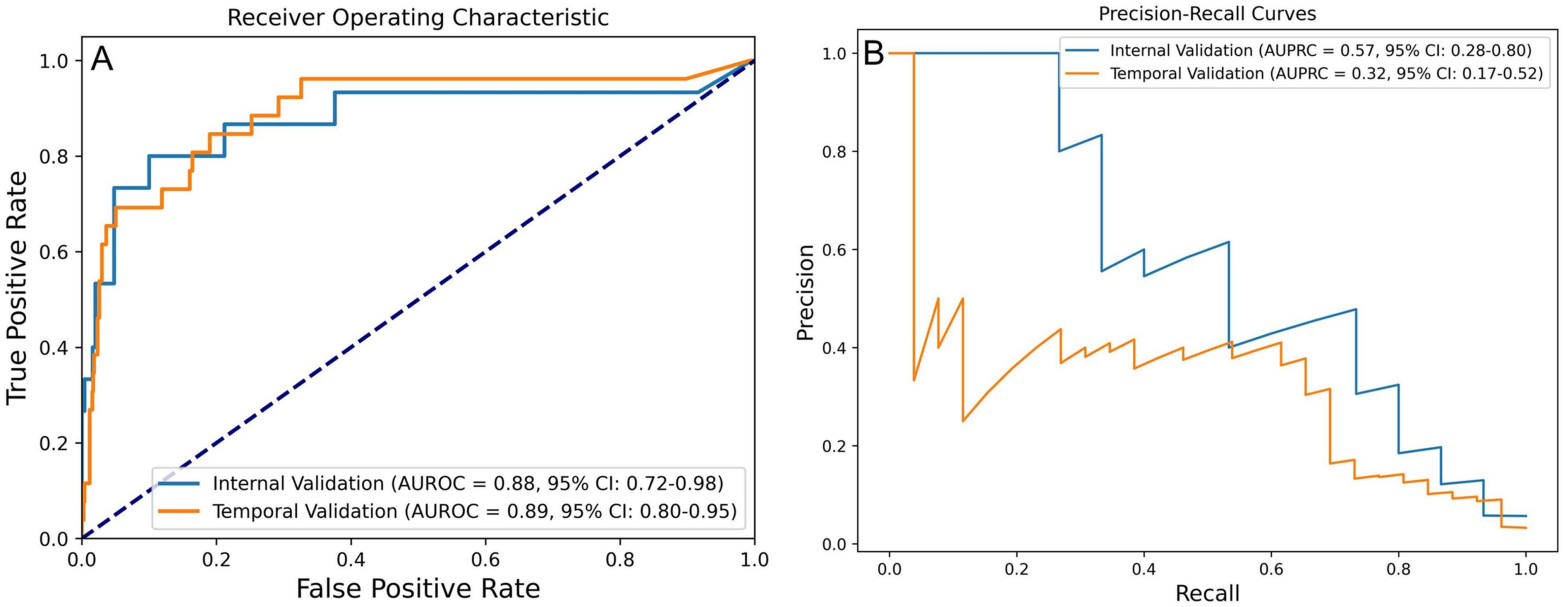
Performance of the GRACE2 score in predicting six-month mortality among AMI patients post-PCI. **(A)** AUROC values of the GRACE2 score. **(B)** AUPRC values of the GRACE2 score.

Internal validation demonstrated an AUROC of 0.88 (95% CI:0.72–0.98) and an AUPRC of 0.57 (95% CI: 0.28–0.80), while temporal validation yielded an AUROC of 0.89 (95% CI: 0.80–0.95) and an AUPRC of 0.32 (95% CI: 0.17–0.52) (Figure 9).

**Figure 10:**
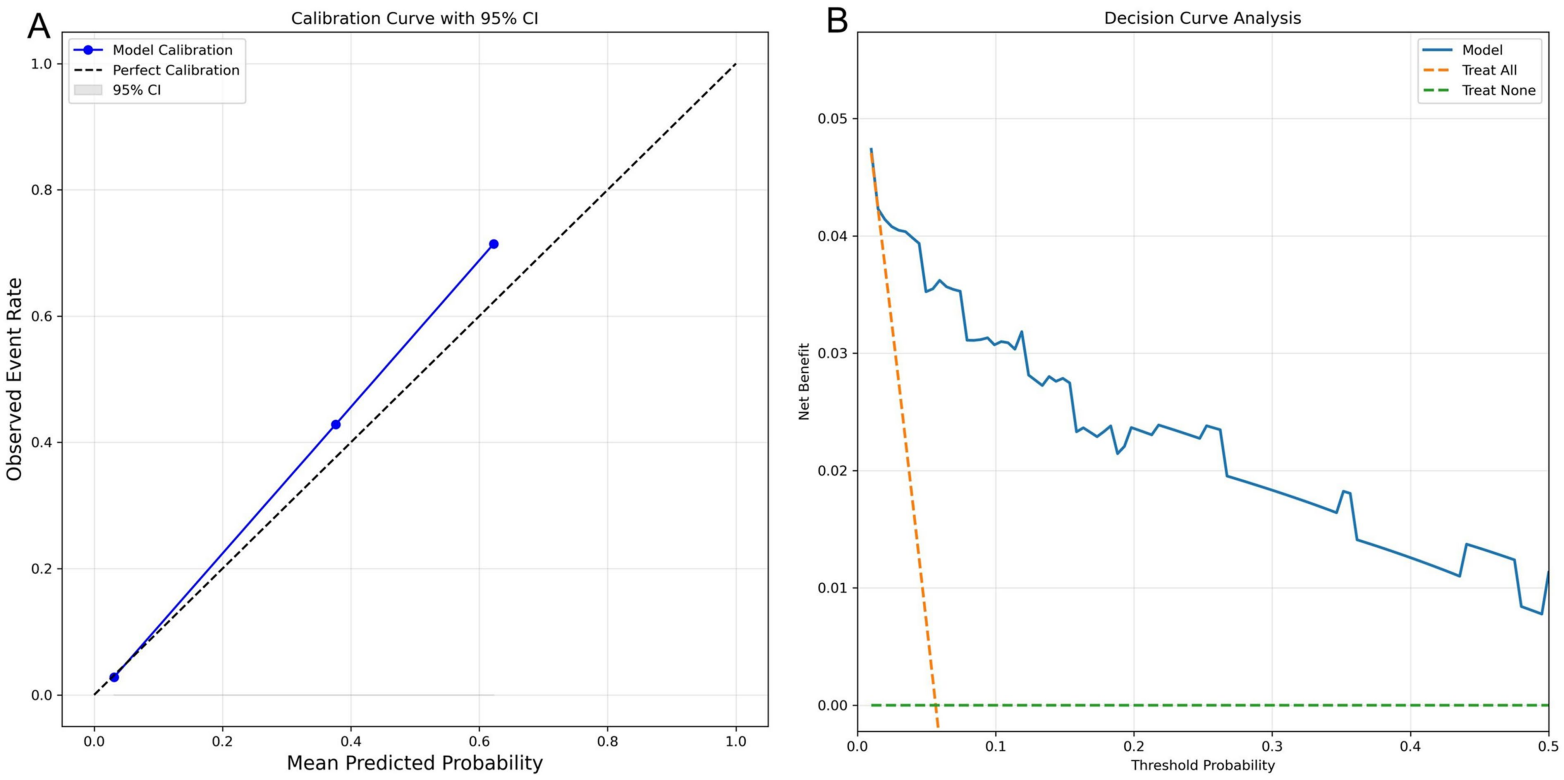
Calibration and decision curve analyses of the GRACE2 score. **(A)** The calibration curve demonstrates the agreement between predicted and observed event probabilities, showing that the model exhibits good calibration across low- to intermediate-risk ranges. **(B)** The DCA evaluates the clinical utility of the model across various threshold probabilities. The prediction model offers a higher net benefit compared to the ‘treat-all’ and ‘treat-none’ strategies across a broad range of threshold probabilities, indicating favorable clinical applicability. DCA, decision curve analysis.

The calibration curve of the GRACE2 score exhibited a strong correlation between predicted and observed risks, particularly at low to intermediate levels (Figure 10A). Additionally, decision curve analysis (DCA) of the GRACE2 score revealed a consistent net benefit when compared to treat-all and treat-none strategies (Figure 10B). These findings underscore the model’s clinical utility for individualized risk stratification in AMI patients post-PCI.

**Figure 11:**
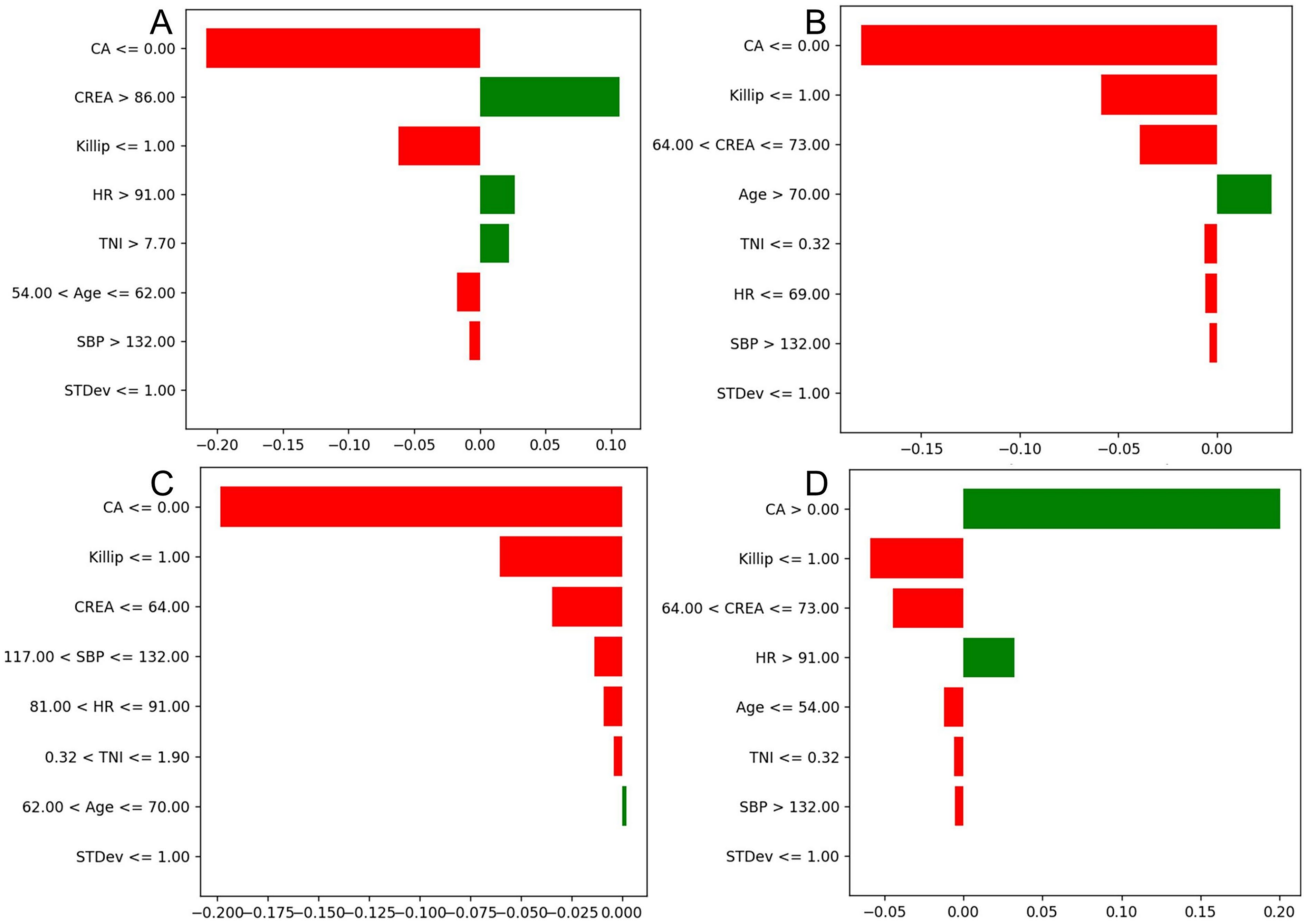
Contribution of key clinical features to individual risk prediction in AMI patients post-PCI. **(A–D)** The directional impact exerted by selected features on model output across distinct patients is illustrated. Horizontal bars denote both the magnitude and direction of contribution, with red indicating a negative (risk-reducing) effect and green indicating a positive (risk-enhancing) effect.

The Local Interpretable Model-Agnostic Explanations (LIME) method was utilized to interpret the GRACE2 score for predicting six-month mortality risk in an individual AMI patient. Figure 11 illustrates the clinical features that most significantly contributed to the risk prediction, thereby facilitating the early identification of key prognostic determinants and enabling timely, personalized therapeutic interventions. Among these four cases, CA was the most influential predictor, followed by Killip class and CREA. Specifically, the absence of CA, Killip class I, and CREA < 73 μmol/L were protective factors, while the presence of CA and CREA > 89 μmol/L were risk-aggravating factors.

**Figure 12:**
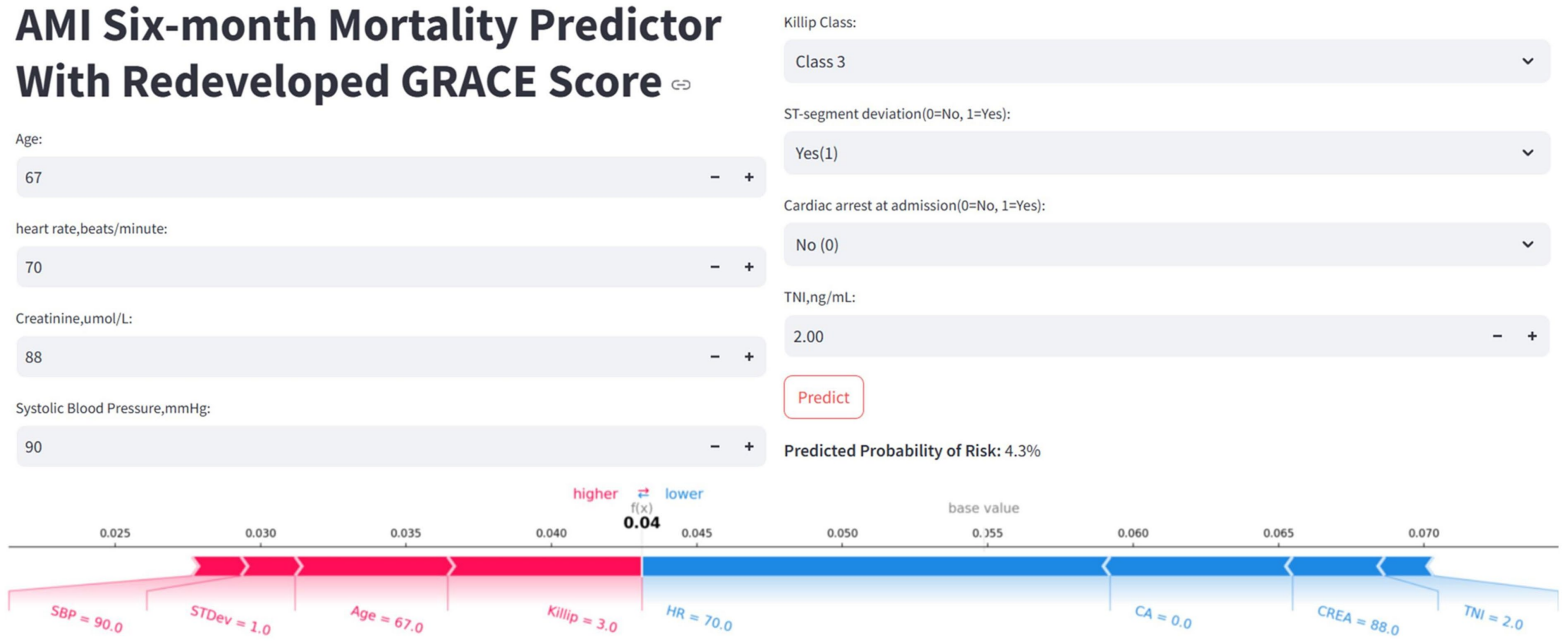
The GRACE2 score for predicting six-month mortality in AMI patients post-PCI. By inputting the eight feature values, the model generates an automatic probability of 4.3% for mortality. The force plot for an individual case visually represents the contributions of each feature, where blue bars on the right indicate factors that increase the likelihood of death, while red bars on the left signify factors that promote survival.

Leveraging the open-source Streamlit Python framework, we developed an interactive web interface that facilitates real-time user interactivity, seamless integration with the prediction model’s data ecosystem, direct deployability into the hospital’s Health Information System, and user-friendliness. Similarly, entering the patient’s parameters yielded a real-time prediction of a 6-month mortality risk of 3.4%. The force plot indicated that Killip class 3, age 67, absence of ST deviation, and SBP of 90 were associated with increased risk, while HR of 70, absence of CA, CREA level of 88, and TNI level of 2 were identified as risk-reducing factors (Figure 12).

## Discussion

The TIMI, GRACE, and ACEF scores are widely utilized prognostic tools for AMI patients, developed sequentially and playing crucial roles in clinical decision-making (Ferenci et al., 2023; Forrest et al., 2023; Gale et al., 2023). This study validated the predictive performance of the GRACE, TIMI, and ACEF scores using local data. The GRACE score achieved an AUROC of 0.84 and an AUPRC of 0.49, outperforming the TIMI score (AUROC: 0.68; AUPRC: 0.19) and the ACEF score (AUROC: 0.69; AUPRC: 0.34). This finding aligns with previous reports (Kumar et al., 2025; Wang et al., 2022; Yanqiao et al., 2022). Subsequently, the original score parameters were utilized as modeling variables, and local data were employed for training. Six ML algorithms were applied to optimize the original scores. The validation of the three classical risk scores using derivation data revealed that the GRACE score exhibited superior predictive performance compared to the ACEF and TIMI scores.

The optimization of these scores through ML algorithms further enhanced their predictive capabilities, indicating that ML methods surpass traditional statistical approaches (Georgiopoulos et al., 2023). Among these, ensemble algorithms demonstrated the highest performance (Liu et al., 2022). Furthermore, ML methods provide superior interpretability and facilitate easier modeling (Sia et al., 2022; Laumer et al., 2022). The GRACE2 and ACEF2 scores displayed significantly better predictive capability than the TIMI2 score, likely due to the incorporation of crucial independent prognostic factors for AMI, such as Killip, LVEF and CREA. These findings suggest that the selection and weighting of modeling parameters are critical determinants of predictive model performance.

The TIMI score, one of the earliest developed risk assessment systems, was established under clinical conditions that differ significantly from the contemporary medical landscape (Gupta et al., 2025). With advancements in medical technology, the mortality risk among AMI patients has markedly decreased, clinical outcomes have improved substantially, and several original risk factors have emerged as key therapeutic targets. These developments have significantly altered the prognosis of AMI, presenting new challenges to the applicability of the TIMI score. Furthermore, the TIMI system employs distinct scoring parameters and rules for STEMI and NSTEMI patients, which adds to its complexity. To investigate the reasons behind the diminished predictive performance of the TIMI score and to evaluate the effectiveness of ML-based optimization, six ML algorithms were employed to refine the original score. The TIMI2 score, which integrates parameters from both STEMI and NSTEMI using ML algorithms, demonstrated enhanced predictive ability, with XGBoost achieving the highest performance (AUROC: 0.75, AUPRC: 0.50). However, this improvement was relatively modest compared to the ACEF2 score, which attained superior performance following RF-based optimization (AUROC: 0.91, AUPRC: 0.51). This discrepancy may be attributed to the variables incorporated in the TIMI score, which diverge from key prognostic factors that influence AMI outcomes, thereby limiting the potential performance enhancements achievable through model optimization.

Conversely, the ACEF score is primarily designed for rapid surgical risk assessment in CAD patients (Lun et al., 2025; Tahir et al., 2025; Wu et al., 2025). The ACEF score was originally developed as a rapid surgical assessment tool for evaluating the clinical prognosis of CAD patients. However, the prognostic factors differ significantly between patients undergoing PCI and those undergoing cardiac surgery. This discrepancy implies that directly applying a model derived for one procedure to assess prognosis in the other may reduce its predictive validity. While contemporary medical progress has considerably improved outcomes relative to the original score development period, this decline is primarily attributable to the limited number of included parameters and substantial changes in the medical environment. Taken together, these findings suggest that the model’s diminished performance reflects both its narrow parameter scope and broader shifts in clinical practice. To align the score with contemporary clinical practice, six ML algorithms were employed to optimize the ACEF model. The results indicated a significant improvement in predictive performance following the optimization, particularly after reallocating parameter weights. This finding suggests that the modeling parameters of the ACEF score remain closely associated with the clinical outcomes of current AMI patients. Among these parameters, LVEF was identified as the most important independent predictor, directly correlating with patient prognosis.

Our local data validation confirmed that the GRACE score exhibits superior predictive ability compared to the other two scores (Georgiopoulos et al., 2023; Hamilton et al., 2024). This finding indicates that the parameters included in the GRACE score remain applicable under current medical and diagnostic conditions. The predictive performance of the redeveloped GRACE score (AUROC: 0.89, AUPRC: 0.55) demonstrated only marginal improvement over the original version (Xin et al., 2025). This suggests that the choice of modeling algorithm is contingent upon the characteristics of the data, and no universally optimal algorithm exists. ML algorithms serve as a complement to traditional modeling methods, delivering better performance and interpretability in scenarios where conventional approaches are inadequate. LR, RF, and DNN exhibited comparable performance with AUROCs of 0.91, 0.89, and 0.77, and AUPRCs of 0.50, 0.58, and 0.41, respectively. This finding somewhat contrasts with previous reports suggesting that ML techniques outperform LR. This discrepancy may be attributed to the absence of data preprocessing steps, such as normalization, prior to applying LR. Ensemble algorithms, exemplified by RF, inherently possess a certain ability to manage data imbalance and normalization, thereby enhancing their modeling performance. Furthermore, interpretability tools such as SHAP can effectively visualize the modeling process and feature importance, further underscoring the advantages of ensemble algorithms. In contrast, the KNN algorithm trains models based on Euclidean distance, which is less relevant to the medical diagnosis process, resulting in inferior performance. Although the DNN algorithm excels at processing multimedia data such as images, audio, and video, its advantages diminish when applied to purely numerical or text-based data (Mahmood et al., 2023, 2024). In the context of developing predictive frameworks utilizing hidden layers and fully connected structures, DNNs are prone to overfitting when used with numerical data and frequently demonstrate unstable performance (Mahmood et al., 2025).

The GRACE score was initially developed for predicting IHM and was subsequently extended to assess longer-term outcomes (Huang et al., 2016; Sia et al., 2022). Our research team conducted a series of studies. In the first study, which included 1,693 AMI patients and analyzed 39 variables, the IHM rate was found to be 2% (Li et al., 2025a). Due to a severe class imbalance, SMOTE was applied, resulting in a final model with 32 predictors. In the second study, involving 1,274 patients and examining 46 variables, the one-year mortality rate was 5.7% (38 out of 1,274), and the final model retained only five predictors (Li et al., 2025b). We discovered that when the proportion of the minority class exceeds 3%, class balancing may be unnecessary and could even increase model complexity (van den Goorbergh et al., 2022). Furthermore, the predictive performance of baseline-data models inevitably declines as the prediction interval lengthens. Notably, the most influential predictors remained largely consistent across models, indicating that their impact on long-term prognosis diminishes after early risk identification and timely intervention. Therefore, dynamic real-time prediction that incorporates longitudinal follow-up data represents a more promising direction. Collectively, these findings underscore that in the era of personalized medicine, the localized application of risk scores may necessitate parameter recalibration based on performance evaluations to maximize predictive utility.

## Limitations

The limitations of this study include: first, the limited sample size and the differences from the original development context of the scores may have adversely affected model performance, as an insufficient sample size can reduce model stability. Second, optimizing the original scores based on the current dataset introduced heterogeneity when compared to optimization using the original derivation data, which limits the persuasiveness of the findings. Third, the suboptimal AUPRC performance of the redeveloped score in validation may be attributed to disruptions in standardized AMI care during the COVID-19 pandemic, including prolonged time to treatment due to COVID-19 screening protocols and increased complexity in managing comorbidities alongside AMI. These deviations from standard care likely reduced the model’s predictive accuracy. Furthermore, the model lacks external validation across diverse geographic settings. The direct impact on the prediction model may result in substantial differences in predictors and their weights, which can lead to decreased model performance during external validation, significant heterogeneity, and diminished generalizability.

## Conclusion and future work

This study validated three classical risk scores, demonstrating that the GRACE score exhibits superior predictive performance compared to the ACEF and TIMI scores for predicting six-month mortality. Furthermore, the redevelopment of these scores through ML techniques significantly enhanced their predictive capabilities. This improvement arises not only from the inherent advantages of ML algorithms but also from the recalibration of parameter weights to align with contemporary clinical practices.

This study identifies several issues that warrant further investigation in future research. First, as witnesses to the evolving landscape of AMI management, the three risk scores reflect the prevailing treatment concepts of their respective eras. Changes in their constituent parameters signify shifts in treatment paradigms. What is required is not merely an update of score weights through novel modeling techniques, but the development of new prediction models by optimizing the score parameters themselves. Second, for risk prediction models initiated at the time of AMI, a significant challenge remains in incorporating subsequent follow-up data to facilitate dynamic risk prediction over varying time intervals, thereby mitigating the progressive decline in predictive performance associated with static baseline data. Third, if risk stratification and subgroup analysis represent alternative forms of individuals sharing certain common characteristics, patients from diverse regions, ethnic groups, and hospitals can also be interpreted as another form of individuality. In this context, whether it remains appropriate to continue pursuing external validation across different time frames and geographical settings warrants further consideration. Fourth, we acknowledge the significance of privacy protection and have implemented data de-identification during the modeling phase. In future work, we will incorporate federated learning and differential privacy techniques to facilitate localized data processing and encrypted parameter sharing. This approach will not only safeguard privacy but also enhance the model’s generalization capability.

## Data Availability

The raw data supporting the conclusions of this article will be made available by the authors, without undue reservation.
