# Supplementary material for: Machine learning redevelopment of GRACE, ACEF, and TIMI scores for 6-month mortality
Source: Front Artif Intell. 2026 Jul 9;9:1838324. doi: 10.3389/frai.2026.1838324 (PMC13391925; doi:10.3389/frai.2026.1838324)
Supplement: Supplementary file 1 [file Data_Sheet_1.docx]

Table S1 GRACE, TIMI, and ACEF Risk Score Parameters and Risk Stratification

| Risk Score | Parameters | Points | Risk Stratification |
| --- | --- | --- | --- |
| GRACE | Age＜30/30～＜40/40～＜50/50～＜60/ 60～＜70/70～＜80/80～＜90/≥90岁 | 0/8/25/41/58/75/91/100 | Low risk≤108/ Intermediate risk 109-140/ High risk＞140 |
|  | Heart Rate＜50/50～＜70/70～＜90/90～＜110/ 110～＜150/150～＜200/≥200次/min | 0/3/9/15/24/38/ 46 |  |
|  | Systolic Blood Pressure＜80/80～99/100～119/120～139/140～159/160～199/≥200 mmHg | 58/53/43/34/24/10/0 |  |
|  | Serum Creatinine0～0.39/0.40～0.79/0.80～1.19/1.20～1.59/1.60～1.99/2.00～3. 99/ ≥4.00 mg/dL | 1/4/7/10/13/21/ 28 |  |
|  | Killip Class：I/II /III/IV | 0/20/39/59 |  |
|  | Cardiac Arrest at Admission | 39 |  |
|  | ST Segment Deviation | 28 |  |
|  | Elevated Cardiac Enzymes | 14 |  |
| TIMI | Age＜65/65～74/＞74 | 1/2/3 | Low risk 0-3/Intermediate risk 4-6/High risk 7-14 |
| (STEMI) | Family history of CAD, hypertension, diabetes, hypercholesterolemia, or history of angina | 1 |  |
|  | Systolic Blood Pressure＜100mmHg | 3 |  |
|  | Heart Rate＞100bmp | 2 |  |
|  | Killip Class：II～IV | 2 |  |
|  | Body Weight＜67kg | 1 |  |
|  | Anterior myocardial infarction or left bundle branch block | 1 |  |
|  | Time to Treatment >4 hours | 1 |  |
| TIMI | Age≥65 | 1 | Intermediate risk 3-4/High risk 5-7 |
| (NSTEMI) | ≥coronary risk factors (e.g., hypertension, diabetes, hyperlipidemia, smoking, family history of premature CAD) | 1 |  |
|  | Prior coronary stenosis ≥50% | 1 |  |
|  | ≥2 angina episodes in the past 24 hours | 1 |  |
|  | ST segment depression ≥0.5 mm | 1 |  |
|  | Elevated cardiac markers | 1 |  |
|  | Aspirin use within 7 days | 1 |  |
| ACEF | Age (years) / LVEF (%) + 1 (if serum creatinine >2 mg/dL) |  | Low risk ≤1/Intermediate risk 1–1.3/High risk >1.3 |

Sample Size Estimation

Although sample size estimation is primarily intended for randomized controlled trials, we conducted a sample size calculation in this study to enhance the credibility of our findings. In the original GRACE score dataset, the 6-month mortality rate among patients with AMI was approximately 4.7%, while the mortality rates in the original ACEF score dataset was 3.3%. In our modeling dataset, the observed 6-month mortality rate was 5.4%, which aligns with the event rates reported in the original studies. Consequently, we set the event rate at 5% for the sample size estimation. Regarding the number of parameters in the risk scores, while the TIMI score comprises a total of 15 parameters (as shown in Table S1), the scoring systems for STEMI and NSTEMI differ. We based our sample size calculation on the STEMI version (8 parameters), which includes more parameters. The detailed calculation is provided below.

Sample Size Calculation for the Redevelopment of GRACE and TIMI Scores

> library(pmsampsize)

> sample_size_binary <- pmsampsize(

+ type = "b",

+ parameters = 8,

+ prevalence = 0.05,

+ nagrsquared = 0.24 # Nagelkerke R²

+ )

> print(sample_size_binary)

NB: Assuming 0.05 acceptable difference in apparent & adjusted R-squared

NB: Assuming 0.05 margin of error in estimation of intercept

NB: Events per Predictor Parameter (EPP) assumes prevalence = 0.05

Samp_size Shrinkage Parameter CS_Rsq Max_Rsq Nag_Rsq EPP

Criteria 1 871 0.900 8 0.079 0.328 0.241 5.44

Criteria 2 465 0.828 8 0.079 0.328 0.241 2.91

Criteria 3 73 0.900 8 0.079 0.328 0.241 0.46

Final 871 0.900 8 0.079 0.328 0.241 5.44

Minimum sample size required for new model development based on user inputs = 871, with 44 events (assuming an outcome prevalence = 0.05) and an EPP = 5.44

Sample Size Calculation for the Redevelopment of ACEF Score

> library(pmsampsize)

> sample_size_binary <- pmsampsize(

+ type = "b",

+ parameters = 3,

+ prevalence = 0.05,

+ nagrsquared = 0.24 # Nagelkerke R²

+ )

> print(sample_size_binary)

NB: Assuming 0.05 acceptable difference in apparent & adjusted R-squared

NB: Assuming 0.05 margin of error in estimation of intercept

NB: Events per Predictor Parameter (EPP) assumes prevalence = 0.05

Samp_size Shrinkage Parameter CS_Rsq Max_Rsq Nag_Rsq EPP

Criteria 1 327 0.900 3 0.079 0.328 0.241 5.45

Criteria 2 175 0.828 3 0.079 0.328 0.241 2.92

Criteria 3 73 0.900 3 0.079 0.328 0.241 1.22

Final 327 0.900 3 0.079 0.328 0.241 5.45

Minimum sample size required for new model development based on user inputs = 327, with 17 events (assuming an outcome prevalence = 0.05) and an EPP = 5.45


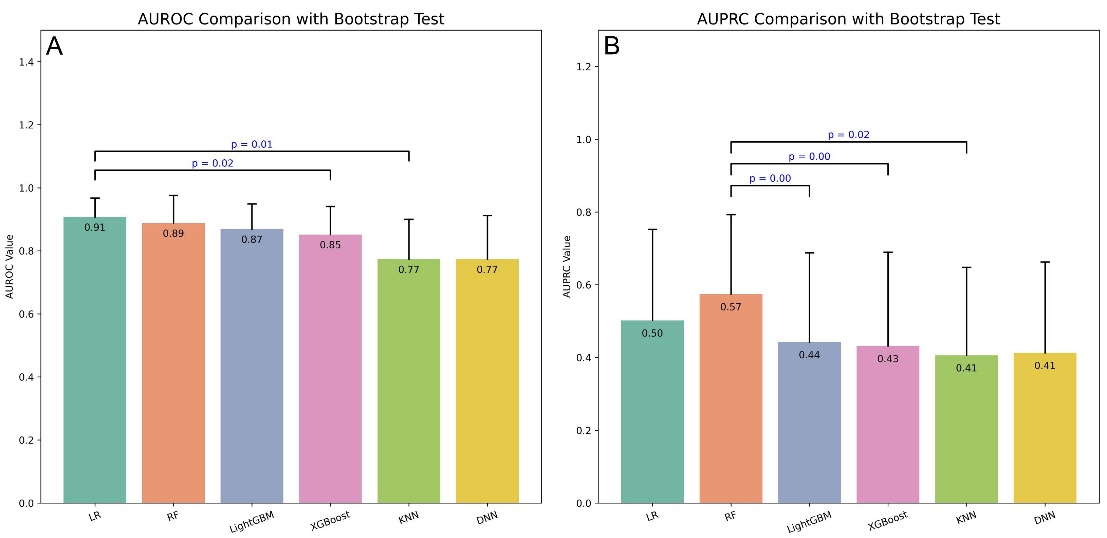


Figure S1. Comparison of redeveloped GRACE scores using bootstrap test. (A) Bootstrap test for AUROC comparison. (B) Bootstrap test for AUPRC comparison. Abbreviations: GRACE, global registry of acute coronary events; RF, random forest; LR, logistic regression; LightGBM, light gradient boosting machine; XGBoost, extreme gradient boosting; KNN, k-nearest neighbors; DNN, deep neural network; AUROC, area under the receiver operating characteristic curve; AUPRC, area under the precision-recall curve.


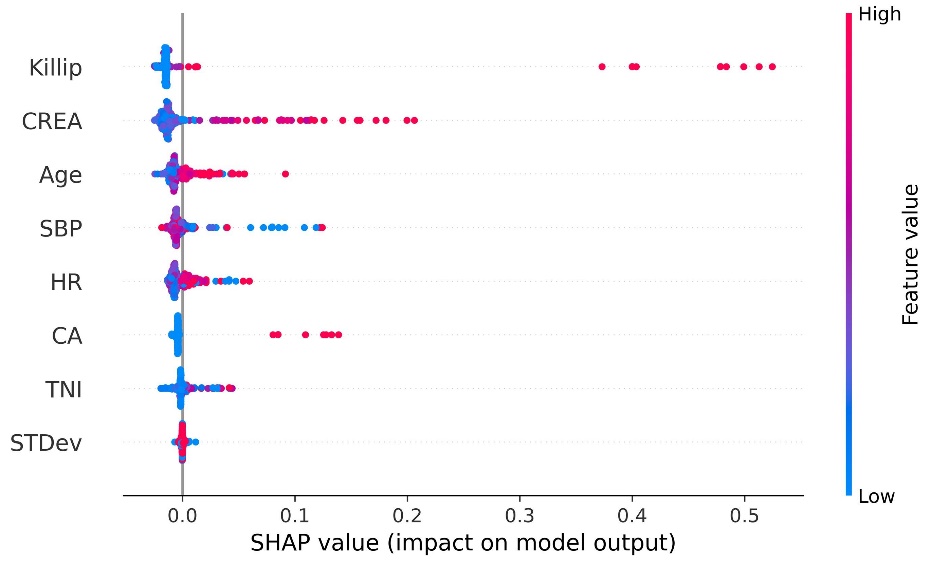


Figure S2: Feature importance scatter plot of the redeveloped GRACE score. Abbreviations: GRACE, global registry of acute coronary events; Killip, killip class; CREA, serum creatinine; SBP, systolic blood pressure; CA, cardiac arrest; HR, heart rate; TNI, troponin I, STDev, ST segment deviation.


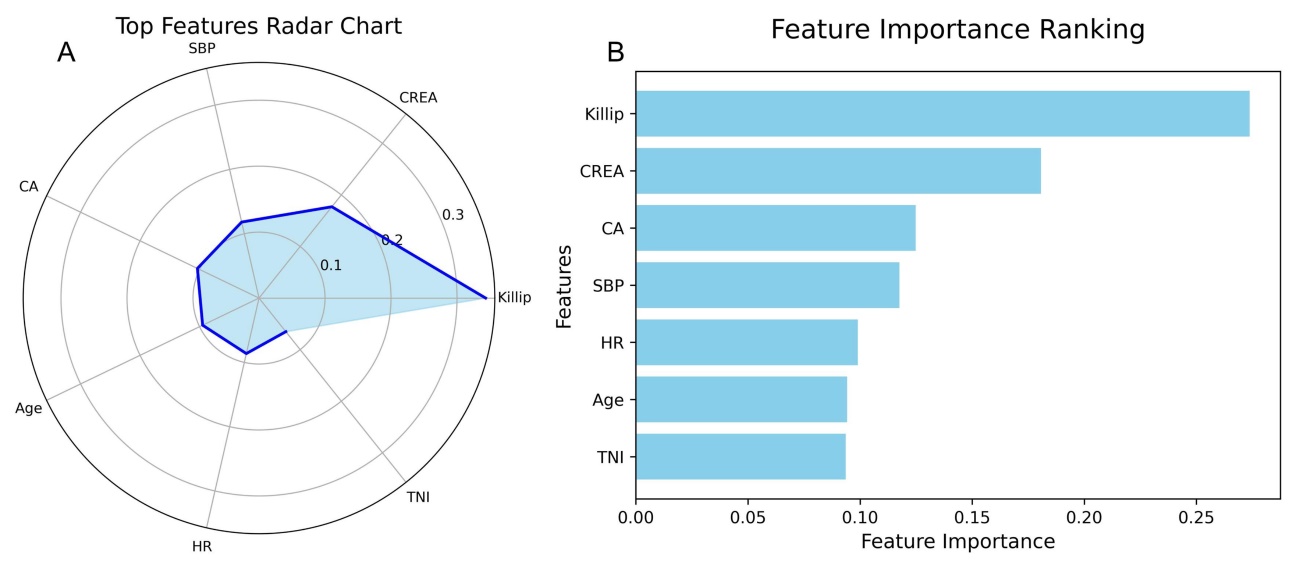


Figure S3: Feature characteristics and importance ranking of the redeveloped GRACE score. (A) Radar chart of model features. (B) Bar plot of feature importance. Abbreviations: GRACE, global registry of acute coronary events; Killip, killip class; CREA, serum creatinine; SBP, systolic blood pressure; CA, cardiac arrest; HR, heart rate.


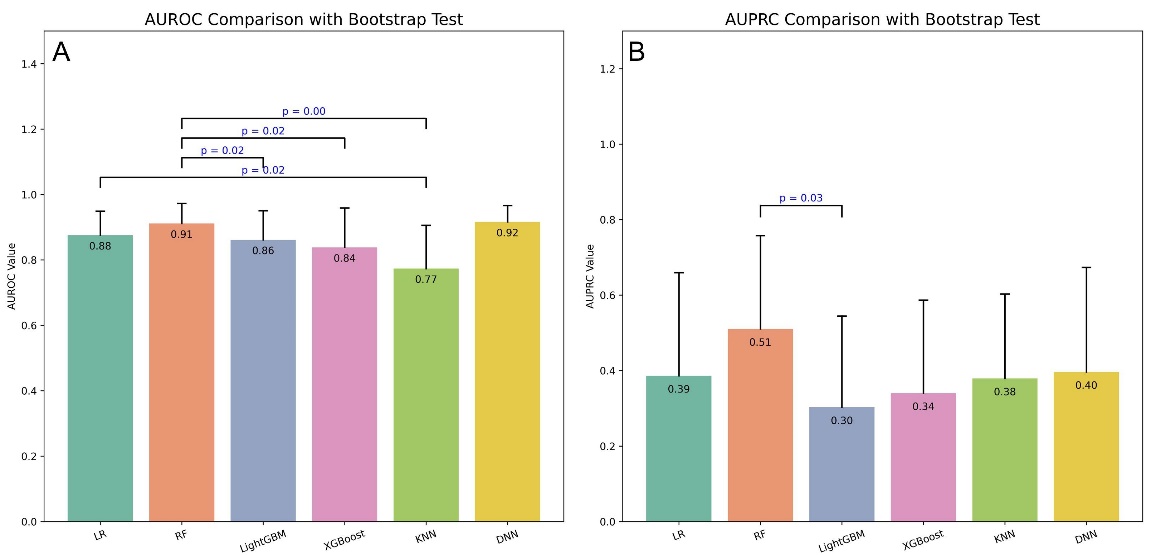


Figure S4: Comparison of redeveloped ACEF scores using bootstrap test. (A) Bootstrap test for AUROC comparison of redeveloped ACEF scores. (B) Bootstrap test for AUPRC comparison of redeveloped ACEF scores. Abbreviations: ACEF, Age-creatinine-ejection fraction; ML, machine learning; RF, random forest; LR, logistic regression; LightGBM, light gradient boosting machine; XGBoost, extreme gradient boosting; KNN, k-nearest neighbors; DNN, deep neural network; AUROC, area under the receiver operating characteristic curve; AUPRC, area under the precision-recall curve.


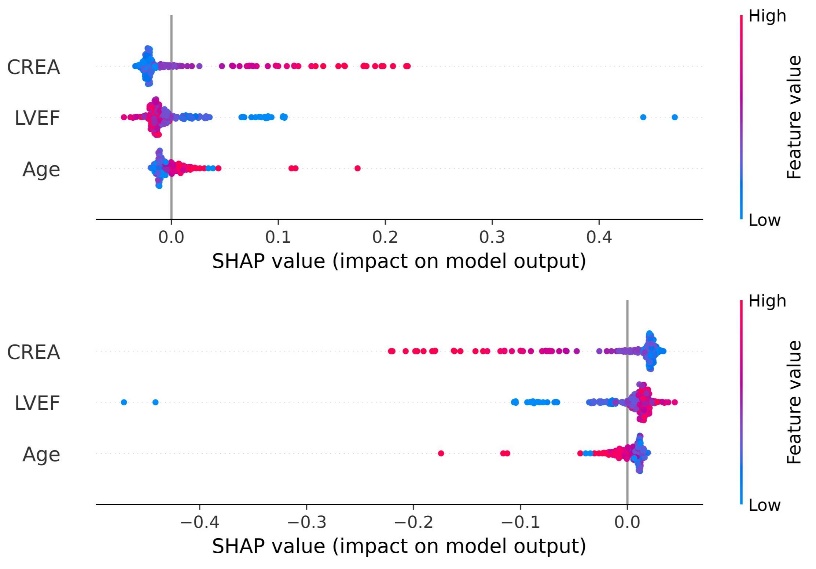


Figure S5: Feature importance scatter plot of the redeveloped ACEF score. Abbreviations: CREA, serum creatinine; LVEF, left ventricular ejection fraction.


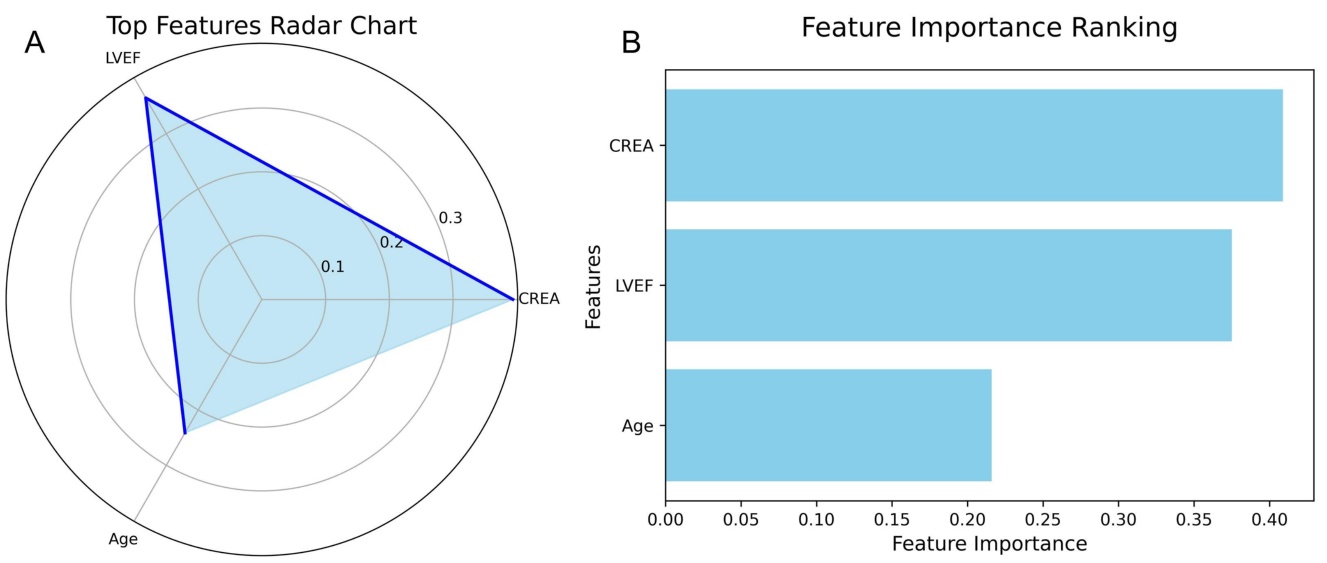


Figure S6: Feature characteristics and importance ranking of the redeveloped ACEF score. (A) Radar chart of model features. (B) Bar plot of feature importance. Abbreviations: ACEF, Age-creatinine-ejection fraction; CREA, serum creatinine; LVEF, left ventricular ejection fraction.


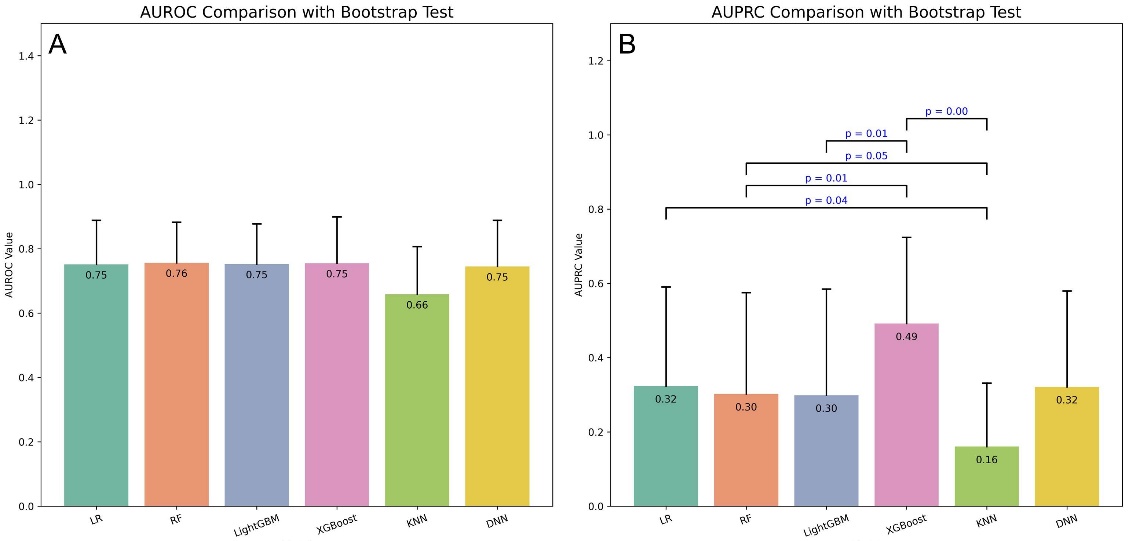


Figure S7: Comparison of redeveloped TIMI scores using bootstrap test. (A) Bootstrap test for AUROC comparison of redeveloped TIMI scores. (B) Bootstrap test for AUPRC comparison of redeveloped TIMI scores. Abbreviations: TIMI, thrombolysis in myocardial infarction; RF, random forest; LR, logistic regression; LightGBM, light gradient boosting machine; XGBoost, extreme gradient boosting; KNN, k-nearest neighbors; DNN, deep neural network; AUROC, area under the receiver operating characteristic curve; AUPRC, area under the precision-recall curve.


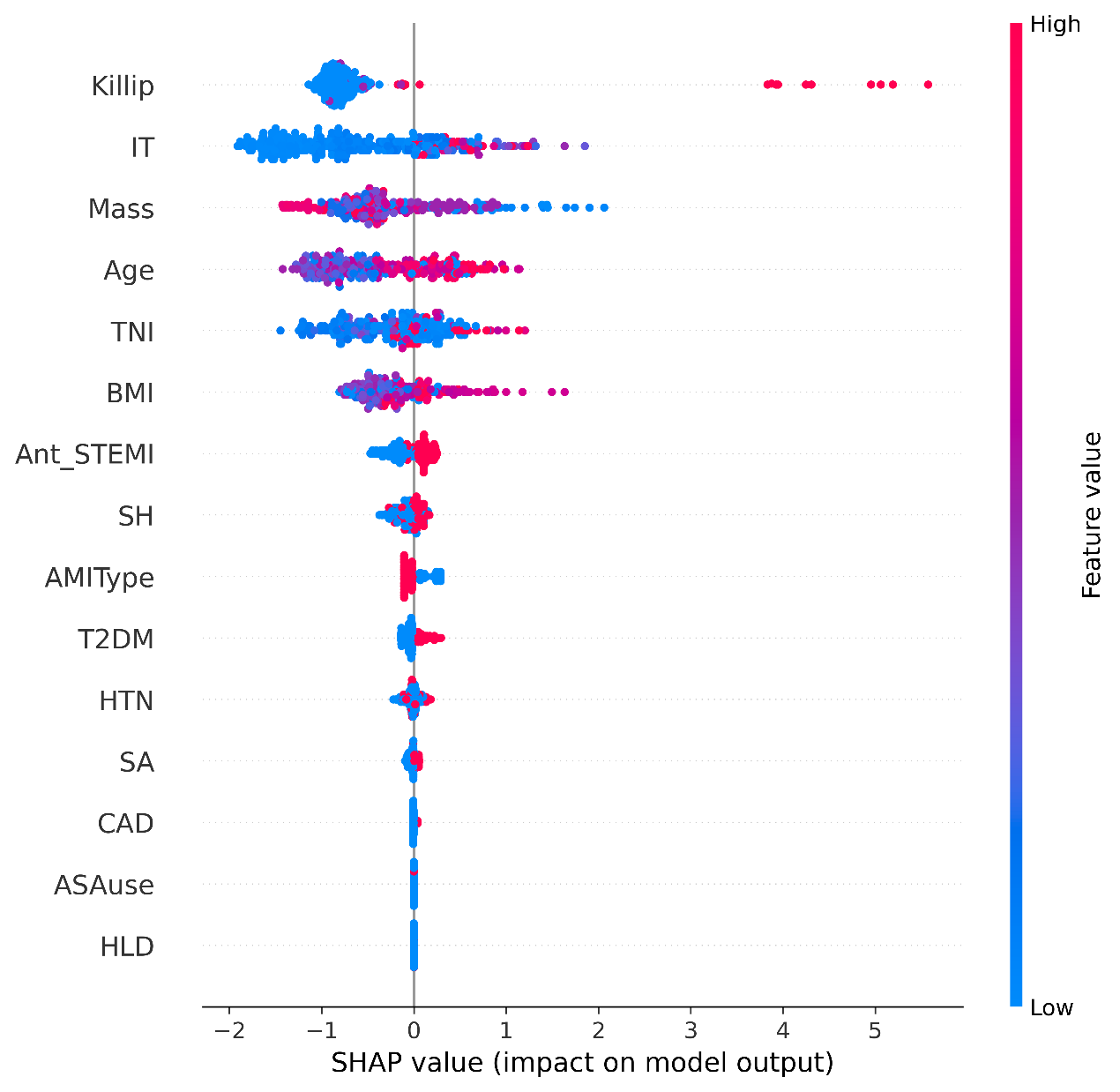


Figure S8: Feature importance scatter plot of the redeveloped TIMI score. Abbreviations: TIMI, thrombolysis in myocardial infarction; Killip: killip class; IT: ischemia time; TNI: troponin I; BMI: body mass index; Ant STEMI: anterior ST segment elevated myocardial infarction; T2DM: type 2 diabetes mellitus; CAD: coronary artery disease; HTN: hypertension; HLD: hyperlipidemia; SH: smoking history; SA: severe angina; ASA use: Aspirin use.


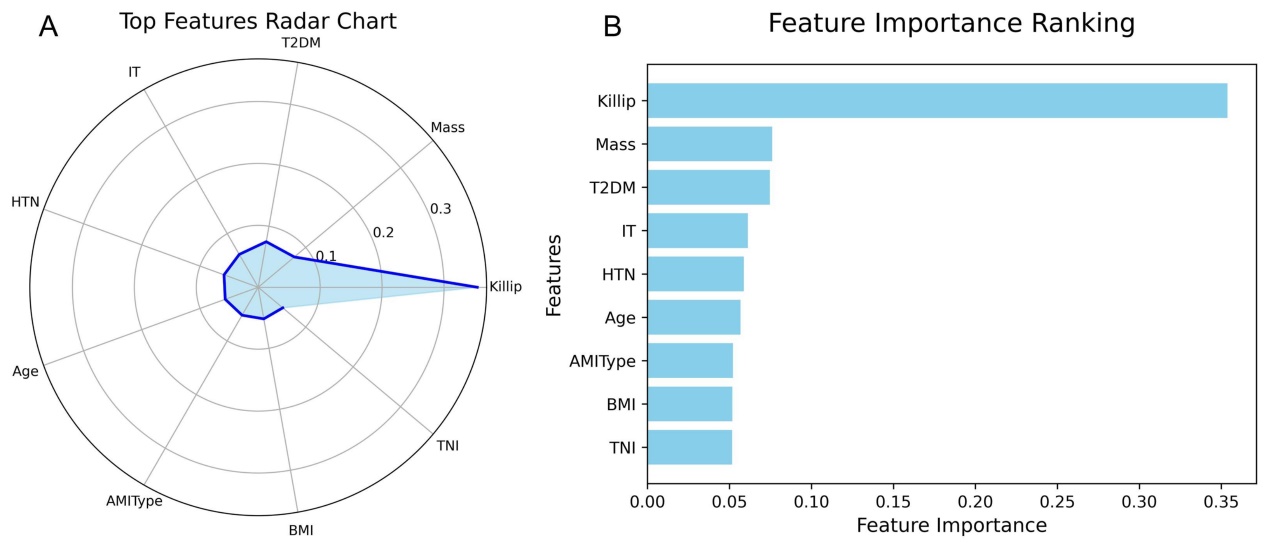


Figure S9: Feature characteristics and importance ranking of the redeveloped TIMI score. (A) Radar chart of model features. (B) Bar plot of feature importance. Abbreviations: TIMI, thrombolysis in myocardial infarction; Killip: killip class; T2DM: type 2 diabetes mellitus; IT: ischemia time; HTN: hypertension; AMI Type: Type of Acute Myocardial Infarction; BMI: body mass index; TNI: troponin I.
